# Needs and Experiences of Users of Digital Navigation Tools for Mental Health Treatment and Supportive Services: Survey Study

**DOI:** 10.2196/27022

**Published:** 2021-06-09

**Authors:** Elizabeth Stafford, Teri Brister, Ken Duckworth, Natali Rauseo-Ricupero, Sarah Lagan

**Affiliations:** 1 The National Alliance on Mental Illness Arlington, VA United States; 2 Beth Israel Deaconess Medical Center Boston, MA United States

**Keywords:** mental health, supportive services, perception, quality, satisfaction, needs, digital tools, directories, navigation

## Abstract

**Background:**

Despite a recent proliferation in web-based and digital resources that are designed to assist users in finding appropriate mental health treatment and supportive services, it can be overwhelming, confusing, and difficult for an individual or family member to access and use an appropriate navigation tool. As digital resources are increasingly sought after, there is an urgent need for a clearer understanding of digital navigation tools in order to help link individuals with the tool that is best suited to their needs.

**Objective:**

The objective of this study was to determine the needs of individuals seeking mental health treatment and supportive services and to quantify their experiences and satisfaction with available digital navigation tools.

**Methods:**

A survey was offered via an email newsletter and social media posting throughout the extended membership of the National Alliance on Mental Illness, which includes both individuals with a mental health condition and their family members and support networks. A 13-item anonymous survey, which consisted of multiple-choice and open response options, was developed to measure participants’ past use of and experiences with web-based, mobile, and phone-based navigation tools. The survey was available from April 9 through May 21, 2020.

**Results:**

A total of 478 respondents completed the survey; the majority of respondents were female (397/478, 83.1%) and aged ≥35 years (411/478, 86%). Younger respondents were more likely to report seeking mental health services for themselves, while older respondents were more likely to be searching for such services on behalf of a family member. The majority of respondents seeking such services on behalf of a family member (113/194, 58.2%) required a combination of mental health treatment and supportive services. Furthermore, two-thirds of respondents (322/478, 67.4%) used a navigation tool to find treatment or services. The majority of respondents who provided feedback about their experiences with navigation tools (224/280, 80%) reported difficulties, with data availability and accuracy being the most commonly reported issues.

**Conclusions:**

The survey results suggest that issues with data availability and accuracy in available navigation tools remain a major barrier for locating timely and appropriate mental health treatment and supportive services within the population of individuals seeking such services. Particularly for individuals seeking care on behalf of a family member, improving the accuracy of and users’ experiences with navigation tools could have a major impact on effectively connecting people to treatment and support services.

## Introduction

Approximately 1 in 5 adults and 1 in 7 adolescents in the United States experience a mental health condition each year [1,2]. Although there has been significant progress in reducing the stigma around seeking care, ensuring parity in insurance coverage, and developing new therapeutic models, many individuals continue to struggle in isolation when attempting to enter and navigate a complex and fractured care system. Each of the over 60 million people with a mental health condition has a unique set of experiences and needs that do not always fit neatly into the array of services that are available to them. Beyond talk therapy, which has become increasingly accessible through advances in telehealth [3], and medication, which can often be prescribed by a primary care physician [4], many people are in immediate need of specialized services, such as crisis care or partial hospitalization programs [5,6]. Additionally, coordinated care for chronic physical health issues [7], housing and legal support, or straightforward guidance and reassurance may be needed as people navigate their overall health care path [8].

In the context of the global COVID-19 pandemic, historic protests against systemic racism and injustice and significantly heightened political tensions, rates of anxiety, depression, and trauma-related stress have skyrocketed [9]. These overlapping crises have brought to light the flaws in our current systems of care. For many individuals, treatment can be inaccessible, unaffordable, and unsuited to their specific needs [1,10,11]. In 2019, approximately 43% of US adults with a mental illness who did not receive needed services stated that they could not afford the cost of treatment, 33% did not know where to go for help, and 20% simply could not afford to invest the necessary time for finding and receiving treatment [1]. For researchers and providers who are invested in reducing this disparity and increasing access to and the appropriate use of mental health services, it is critical to understand how people currently seek out and access treatment and support options.

Health care–focused technology solutions have proliferated in recent years. A recent study of downloadable apps for personal mental health management and support reported more than 1400 individual products in the Google Play and iTunes stores [12]. Insurance providers who offer Affordable Care Act marketplace and Medicare Advantage plans are required to publish provider directories [13,14], and many insurers and health systems offer web-based provider search and appointment scheduling tools. However, successfully connecting to appropriate services can be challenging, even with the aid of a directory or search tool. Users may be unsure about what service is the most appropriate for their specific needs and how to parse information on the internet about a diversity of specialties and treatment modalities with overly technical names and descriptors [15]. Provider directories are prone to inaccurate or out-of-date information [16]. Mental health providers are less likely to participate in insurance network plans and, consequently, may change their offerings and availability at will [17]. Amid a flood of information, users who are likely already experiencing considerable stress may be overwhelmed and left without clear guidance.

Current efforts for simplifying this search process and supporting user decision making include the development of e-hubs—web-based directories of local resources and community services that enable users to efficiently search for and organize potential treatment and support options. With the recent rise in web-based mental health tools, more comprehensive e-hubs have emerged to support access to web-based resources such as web-based forums, self-help videos, and support groups [18,19]. It is particularly relevant that these web-based navigation resources and e-hubs have been community [19] and expert driven [18]. Although the availability and complexity of these navigation tools has increased, no single e-hub is recognized as a gold-standard model.

The COVID-19 pandemic has highlighted the importance of accessible mental health services [20] and has resulted in a surge in the demand for web-based treatment options. Despite the clear potential that digital navigation tools have for improving accessibility to both web-based and in-person resources, research on this topic is sparse and often limited to the experiences of younger adults [21]. Continued progress requires a more complete understanding of how people search for mental health treatment and related services and how satisfied they are with their experiences of using different tools. Thus, in this survey study, we aimed to assess how people seeking mental health resources use and perceive technology-based navigation resources. As we were aware of prior research suggesting that information on interventions is the most important [22] and that older adults are becoming increasingly comfortable with operating web-based platforms [23], we hypothesized that overall satisfaction with digital navigation tools would be high. However, given the diversity of the needs and experiences of users and the difficulty of maintaining accurate informational resources across a variety of platforms, quantifying the actual experience of users is critical.

## Methods

The National Alliance on Mental Illness (NAMI) is a nonprofit, national advocacy group based in the United States. By using a nationwide network of state and local community–based affiliates, NAMI provides education, awareness, and advocacy programs to support the mission of empowering individuals with mental illness and their family members to lead productive and fulfilling lives. An integral part of this work is the operation of the NAMI HelpLine—an information and support center that is staffed by individuals with lived experiences of mental illness. The HelpLine served over 150,000 individuals in 2019 through phone calls, emails, social media interactions, web-based resources, and letters [24]. Throughout the first half of 2020, the HelpLine received a record number of inquiries and requests, which were in large part due to concerns about COVID-19 and stress-related mental health concerns.

In order to better serve the needs of the community and improve service delivery, NAMI conducted a web-based, anonymous survey to assess individuals’ needs and experiences when searching for mental health resources. This voluntary survey was promoted through NAMI’s national leadership email newsletter and the NAMI Facebook and Twitter accounts. The survey was available from April 9 through May 21, 2020. The eligibility requirements included any adult (aged ≥18 years) located in the United States who had searched for mental health treatment or services for themselves or someone else via the internet.

The survey consisted of 13 questions, including demographic questions, questions abouts the types of mental health services sought, questions about the types of tools used, and questions about prior experience with navigation tools. Multimedia Appendix 1 outlines the questions that were asked in the survey as well as the type of responses available.

## Results

A total of 520 individuals completed the survey, and 478 individuals met the eligibility requirements. A large portion of respondents (397/478, 83.1%) were female, 14.6% (70/478) were male, and 1.5% (7/478) were gender nonbinary or self-described their gender identity. Half of the respondents were aged ≥55 years (242/478, 50.6%), 35.4% (169/478) of individuals were aged 35-54 years, and 13.4% (64/478) of participants reported that they were between the ages of 18 and 34 years. Most participants (403/478, 84.3%) self-identified as White or Caucasian, 6.3% (30/478) of participants self-identified as Black or African American, 2.5% (12/478) reported a self-described identity, 2.1% (10/478) self-identified as mixed or multiracial, 1.7% (8/478) self-identified as Asian or Pacific Islander, and 1% (5/478) self-identified as Native American or Alaska Native. Demographics are detailed in Table 1.

**Table 1:** Demographic characteristics of participants.

Characteristics of survey respondents		Values, n (%)
**Gender**		
	Female	397 (83.1)
	Male	70 (14.6)
	Nonbinary or self-described	7 (1.5)
	Unreported	4 (0.8)
**Race and ethnicity**		
	Asian or Pacific Islander	8 (1.7)
	Black or African American	30 (6.3)
	Native American or Alaska Native	5 (1)
	White or Caucasian	403 (84.3)
	Mixed or multiracial	10 (2.1)
	Self-described	12 (2.5)
	Unreported	10 (2.1)
**Age category (years)**		
	18-34	64 (13.4)
	35-54	169 (35.4)
	≥55	242 (50.6)
	Unreported	3 (0.6)

A total of 67.4% (322/428) of respondents reported that they used a web-based platform, phone-based directory, or mobile app to find mental health services. Older adults were less likely to use navigation tools compared to younger adults, with 37.2% (90/242) of those aged ≥55 years reporting that they had never used a web-based platform, phone-based directory, or mobile app to find mental health services, as shown in Table 2. Younger respondents (aged 18-34 years) were less likely to seek support by using the phone-based HelpLine or a phone-based directory compared to middle- and older-aged individuals. While 21.9% (90/411) of respondents aged ≥35 years reported seeking resources by using the phone-based HelpLine or a phone-based directory, only 7.8% (5/64) of younger participants used a phone-based directory, and 12.5% (8/64) of younger participants used a mobile app to seek resources. Younger respondents (55/64, 85.9%) and middle-aged respondents (aged 35-54 years; 115/169, 68%) were more likely to report searching for resources for themselves, while older individuals (aged ≥55 years) were more likely to report searching for resources on behalf of a family member or another individual who required support (131/242, 54.1%).

**Table 2:** Use of navigation tools by age.

Responses		All respondents	Respondents aged 18-34 years	Respondents aged 35-54 years	Respondents aged ≥55 years
**Tool sought and used, n (%)**					
	Web-based search platform	289 (60.4)	45 (70.3)	112 (66.3)	131 (54.1)
	Mobile app	48 (10)	8 (12.5)	18 (10.7)	22 (9.1)
	Phone-based HelpLine or directory	97 (20.3)	5 (7.8)	38 (22.5)	52 (21.5)
No tool used, n (%)		156 (32.6)	17 (26.6)	48 (28.4)	90 (37.2)
Seeking on behalf of self, n (%)		281 (58.8)	55 (85.9)	115 (68)	111 (45.9)
Seeking on behalf of another, n (%)		197 (41.2)	9 (14.1)	54 (32)	131 (54.1)
Total responses, n		478	64	169	242

**Figure 1:**
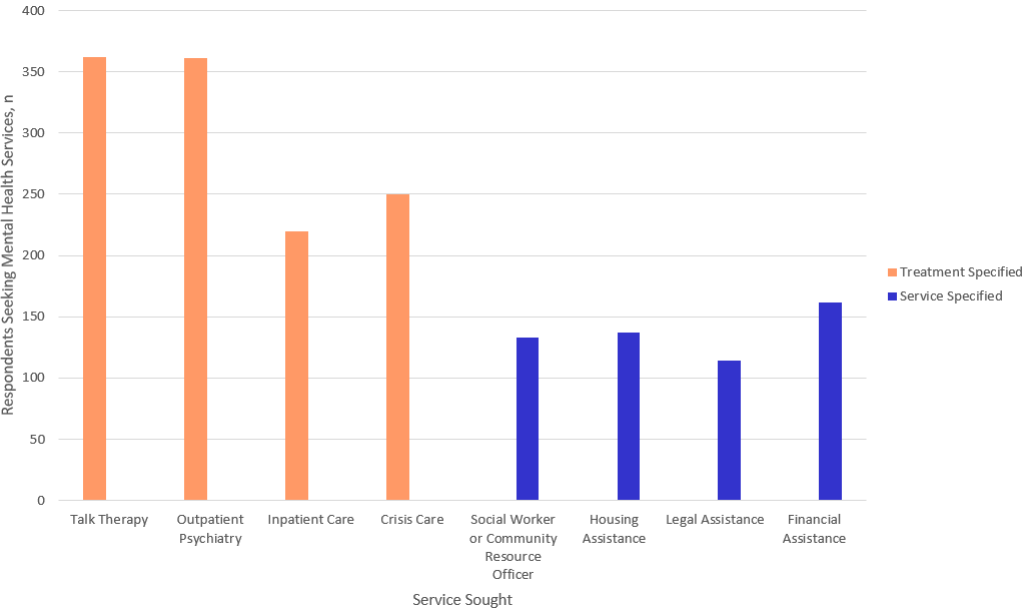
The most common services sought by survey respondents.

**Figure 2:**
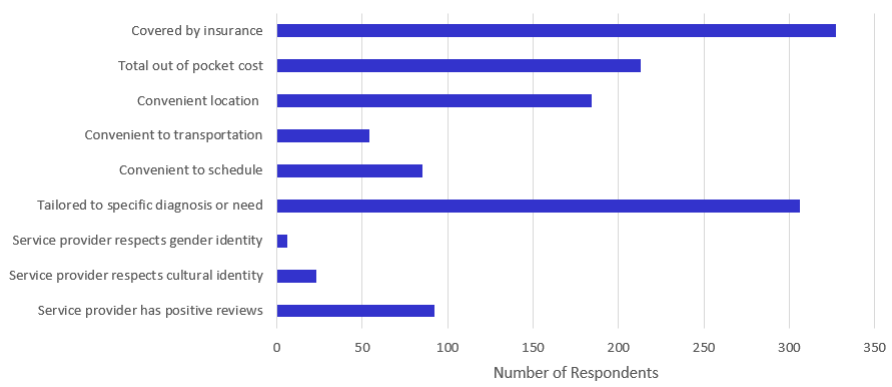
The most common concerns among survey respondents.

In total, 48.2% (228/473) of respondents reported only seeking resources that were related to treatment (talk therapy, outpatient psychiatry, inpatient care, or crisis care), while 49.9% (236/473) reported seeking a combination of treatment and support resources (social worker or community resource officer, housing, and legal or financial assistance). Across all age groups, those who were seeking resources on behalf of someone else were the most likely to be seeking a combination of treatment and supportive services (113/194, 58.2%; Table 3). The top three resources that respondents reported seeking were talk therapy (362/473, 76.5%), outpatient psychiatry (361/473, 76.3%), and crisis care (250/473, 52.9%), as depicted in Figure 1. The highest rated concern reported was whether a service was covered by their insurance. The lowest rated concerns, which are illustrated in Figure 2, included transportation, cultural considerations, and respect for gender identity.

**Table 3:** Type of service sought among individuals seeking services on behalf of themselves versus those seeking services on behalf of another.

Responses	All respondents	Seeking on behalf of self	Seeking on behalf of another
Total responses, n	473	279	194
Treatment only, n (%)	228 (48.2)	151 (54.1)	77 (39.7)
Support service only, n (%)	9 (1.9)	5 (1.8)	4 (2)
Combination of treatment and support services, n (%)	236 (49.9)	123 (44)	113 (58.2)

Of the 280 survey respondents who provided details about their experiences with navigation tools, 224 (80%) indicated that they had experienced frustration or difficulty. The most commonly reported difficulties were about data availability and quality; approximately half of respondents who reported experiencing issues with navigation tools indicated that their search results did not contain enough information (118/280, 42.1%) or that the information provided was incorrect or out of date (132/280, 47.1%), as detailed in Table 4. Older adults who reported using navigation tools (103/132, 78%) were not significantly more likely than younger age groups (31/42, 74%) to report difficulties (*P*=.57), but participants who were seeking services on behalf of a family member or someone else (89/106, 84%) were somewhat more likely to experience issues compared to those seeking services for themselves (135/174, 77.6%; Table 5). However, the difference was not statistically significant (*P*=.20). For individuals playing caregiver roles, the availability of comprehensive and user-friendly navigation tools is especially relevant, as the services that are needed tend to be more complex in nature.

**Table 4:** Difficulties with navigation tools by age.

Responses		All respondents	Respondents aged 18-34 years	Respondents aged 35-54 years	Respondents aged ≥55 years
Provided details about the use of a navigation tool, n		280	42	104	132
**Any difficulty, n (%)**		224 (80)	31 (73.8)	88 (84.6)	103 (78)
	Tool was confusing	50 (17.9)	8 (19)	21 (20.2)	21 (15.9)
	Specific need was not covered	95 (33.9)	14 (33.3)	37 (35.6)	43 (32.6)
	Geographic area was not covered	53 (18.9)	7 (16.7)	21 (20.2)	24 (18.2)
	Not enough information provided	118 (42.1)	22 (52.4)	45 (43.3)	51 (38.6)
	Information provided was incorrect or out of date	132 (47.1)	25 (59.5)	47 (45.2)	59 (44.7)
No difficulty, n (%)		56 (20)	11 (26.2)	16 (15.4)	29 (22)

**Table 5:** Difficulties with navigation tools among individuals seeking services on behalf of themselves versus those seeking services on behalf of another.

Responses		Seeking on behalf of self	Seeking on behalf of another
Provided details about the use of a navigation tool, n		174	106
**Any difficulty, n (%)**		135 (77.6)	89 (84)
	Tool was confusing	32 (18.4)	18 (17)
	Specific need was not covered	54 (31)	41 (38.7)
	Geographic area was not covered	31 (17.8)	22 (20.8)
	Not enough information provided	72 (41.4)	46 (43.4)
	Information provided was incorrect or out of date	81 (46.6)	51 (48.1)
No difficulty, n (%)		39 (22.4)	17 (16)

## Discussion

The results from our web-based survey showed that two-thirds (322/478, 67.4%) of adults seeking mental health treatment or support services used web-based, phone, and app-based tools to find resources and information, but the majority (224/280, 80%) experienced difficulties and dissatisfaction largely due to out-of-date and incorrect information. The use of these tools varied with age, with younger people being more likely to use navigation tools, especially web-based platforms or mobile apps, and more likely to seek mental health services for themselves as opposed to seeking such services for someone else.

The survey results suggest that existing digital navigation resources do not meet the demands of users. Although widespread internet access and increased comfort with web-based tools has resulted in the increased visibility of web-based mental health resources, a lack of reliable curation has led to an accumulation of out-of-date and incorrect information. These results suggest that a focus on the quality of content may be the most important next step in this research area. Offering some degree of personalization in insurance coverage matching services also appears to be a crucial factor—one that is not commonly available to date. Furthermore, web-based resources must account for the different needs and priorities within user populations. For instance, a previous study suggested that older adults (aged ≥65 years) were more willing to engage with digital tools after receiving digital skills training and a demonstration of a tool’s value [25]. However, an alternative study found significant variance in older individuals’ (aged ≥65 years) use and perceptions of the benefits of digital tools; the differences were more aligned with racial identity and socioeconomic status than with age [26]. The results from the survey conducted in our study did not show a meaningful difference in the percentages of older adults who found that navigation tools were confusing to use.

The primary limitation of this study lies in the generalizability of the results. As the survey was conducted via a web-based platform and promoted through digital communication channels, it can be assumed that all participants had reliable internet access and at least a moderate level of comfort with using the internet. Participants with a connection to NAMI may have been more likely than the general population to have past experiences with seeking mental health services and support, and many (eg, those seeking inpatient treatment) were likely to be individuals or family members of an individual with a serious mental illness. Our findings may consequently have limited generalizability to individuals seeking services and support for less intensive and more common mental health conditions. The participant population was heavily weighted toward older White females, indicating that issues affecting members of minority communities are underrepresented. The survey did not collect information on socioeconomic status or geographic location, of which both are factors that may significantly influence users’ needs for and experiences with seeking services. Finally, this study was conducted from April to May 2020 (ie, early stage of the COVID-19 pandemic), and we expect that the concerns and experiences of those seeking mental health care may have changed, as the pressures and constraints of the ongoing pandemic have continued to affect both mental health needs and the accessibility of related services. Finally, this study was only offered in English; therefore, it is not representative of many patients and families who read in other languages.

In summary, there remains a need for improved digital navigation resources and e-hubs, as existing services do not meet the needs and expectations of users. As more mental health resources move to web-based platforms, ensuring that services remain easily searchable and accessible will only become more important.

## Appendix

Multimedia Appendix 1 Finding mental health services: Survey on experiences and needs.

## Abbreviations

NAMI: National Alliance on Mental Illness

## References

[1] (2020). 2019 NSDUH Annual National Report. Substance Abuse and Mental Health Services Administration (SAMHSA).

[2] Whitney DG, Peterson MD (2019). US national and state-level prevalence of mental health disorders and disparities of mental health care use in children. JAMA Pediatr.

[3] Abrams Z (2020). How well is telepsychology working?. Monitor on Psychology.

[4] Beck AJ, Page C, Buche J, et al (2019). Behavioral health service provision by primary care physicians. University of Michigan Behavioral Health Workforce Research Center.

[5] (2020). National Mental Health Services Survey (N-MHSS): 2019, data on mental health treatment facilities. Substance Abuse and Mental Health Services Administration (SAMHSA).

[6] Wiess AJ, Barrett ML, Heslin KC, Stocks C (2016). Trends in emergency department visits involving mental and substance use disorders, 2006-2013. Agency for Healthcare Research and Quality (AHRQ), Healthcare Cost Utilization Project (HCUP).

[7] Panagioti M, Bower P, Kontopantelis E, Lovell K, Gilbody S, Waheed W, Dickens C, Archer J, Simon G, Ell K, Huffman JC, Richards DA, van der Feltz-Cornelis C, Adler DA, Bruce M, Buszewicz M, Cole MG, Davidson KW, de Jonge P, Gensichen J, Huijbregts K, Menchetti M, Patel V, Rollman B, Shaffer J, Zijlstra-Vlasveld MC, Coventry PA (2016). Association between chronic physical conditions and the effectiveness of collaborative care for depression: An individual participant data meta-analysis. JAMA Psychiatry.

[8] Koh HK, Rudd RE (2015). The arc of health literacy. JAMA.

[9] Czeisler MÉ, Lane RI, Petrosky E, Wiley JF, Christensen A, Njai R, Weaver MD, Robbins R, Facer-Childs ER, Barger LK, Czeisler CA, Howard ME, Rajaratnam SMW (2020). Mental health, substance use, and suicidal ideation during the COVID-19 pandemic - United States, June 24-30, 2020. MMWR Morb Mortal Wkly Rep.

[10] Ofonedu ME, Belcher HME, Budhathoki C, Gross DA (2017). Understanding barriers to initial treatment engagement among underserved families seeking mental health services. J Child Fam Stud.

[11] Gulliver A, Griffiths KM, Christensen H (2010). Perceived barriers and facilitators to mental health help-seeking in young people: a systematic review. BMC Psychiatry.

[12] Larsen ME, Huckvale K, Nicholas J, Torous J, Birrell L, Li E, Reda B (2019). Using science to sell apps: Evaluation of mental health app store quality claims. NPJ Digit Med.

[13] (2015). Final 2016 Letter to Issuers in the Federally-facilitated Marketplaces. Centers for Medicare and Medicaid Services.

[14] (2015). Advance Notice of Methodological Changes for Calendar Year (CY) 2016 for Medicare Advantage (MA) Capitation Rates, Part C and Part D Payment Policies and 2016 Call Letter. Centers for Medicare and Medicaid Services.

[15] Dahlke DV, Fair K, Hong YA, Beaudoin CE, Pulczinski J, Ory MG (2015). Apps seeking theories: results of a study on the use of health behavior change theories in cancer survivorship mobile apps. JMIR Mhealth Uhealth.

[16] Adelberg M, Frakt A, Polsky D, Strollo MK (2019). Improving provider directory accuracy: can machine-readable directories help?. Am J Manag Care.

[17] Davenport S, Gray TJ, Melek SP (2019). Addiction and mental health vs. physical health: Widening disparities in network use and provider reimbursement. Milliman Research Report.

[18] Hsiung RC (2004). The best of both worlds: An online self-help group hosted by a mental health professional. Cyberpsychol Behav.

[19] Bennett K, Reynolds J, Christensen H, Griffiths KM (2010). e-hub: an online self-help mental health service in the community. Med J Aust.

[20] Liu S, Yang L, Zhang C, Xiang YT, Liu Z, Hu S, Zhang B (2020). Online mental health services in China during the COVID-19 outbreak. Lancet Psychiatry.

[21] Pretorius C, Chambers D, Coyle D (2019). Young people's online help-seeking and mental health difficulties: Systematic narrative review. J Med Internet Res.

[22] Wetterlin FM, Mar MY, Neilson EK, Werker GR, Krausz M (2014). eMental health experiences and expectations: a survey of youths' Web-based resource preferences in Canada. J Med Internet Res.

[23] Anderson M, Perrin A (2017). Tech adoption climbs among older adults. Pew Research Center.

[24] NAMI 2019 Annual Report. National Alliance on Mental Illness (NAMI).

[25] Schreurs K, Quan-Haase A, Martin K (2017). Problematizing the digital literacy paradox in the context of older adults’ ICT use: Aging, media discourse, and self-determination. Canadian Journal of Communication.

[26] Cotten SR, Francis J, Kadylak T, Rikard RV, Huang T, Ball C, DeCook J (2016). A tale of two divides: Technology experiences among racially and socioeconomically diverse older adults. Human Aspects of IT for the Aged Population.

