# Supplementary material for: Needs and Experiences of Users of Digital Navigation Tools for Mental Health Treatment and Supportive Services: Survey Study
Source: JMIR Ment Health. 2021 Jun 9;8(6):e27022. doi: 10.2196/27022 (PMC8241433; doi:10.2196/27022)
Supplement: Multimedia Appendix 1 [file mental_v8i6e27022_app1.doc]

| **Question** | **Response Options** |
| --- | --- |
| Do you currently live in the United States? | 1. Yes 2. No |
| What is your age? | 1. 17 or younger 2. 18 – 25 3. 26 – 34 4. 35 – 44 5. 45 – 54 6. 55 – 64 7. 65 or older 8. Prefer not to answer |
| What is your gender? | 1. Male 2. Female 3. Non-binary 4. Prefer not to answer 5. Another identity (please specify): ______________ |
| What is your race/ethnic background? | 1. Asian/Pacific Islander 2. Black/African American 3. Native American/Alaska Native 4. White/Caucasian 5. Mixed/Multiracial 6. Prefer not to answer 7. Another background (please specify): ____________ |
| Are you of Hispanic, Latinx, or Spanish origin or descent? | 1. Yes 2. No 3. Prefer not to answer |
| Have you ever searched for mental health treatment or related services, like social services or financial assistance? Please select all that apply. | 1. Yes, I have searched for services for myself 2. Yes, I have searched for services for my family member 3. Yes, seeking services for someone else 4. No, I have never searched for mental health treatment or related services |
| When you searched for treatment or services, what type of services were you seeking? Please select all that apply. | 1. Talk therapy 2. Outpatient psychiatry 3. Inpatient care 4. Crisis care 5. Social worker or community resource officer 6. Housing assistance 7. Legal assistance 8. Financial assistance 9. Another resource (please specify): ________________ |
| When you search for treatment or services, what factors are most important to help you decide? Please select up to three. | 1. The service is covered by my insurance 2. The out of pocket cost of the service is affordable 3. The service is convenient to my location 4. The service is convenient to my transportation options 5. The service is offered within my schedule / availability 6. The service is appropriate for my specific needs / diagnosis 7. The provider will respect and understand my gender identity 8. The provider will respect and understand my cultural identity 9. The service or provider has positive consumer reviews or satisfaction ratings 10. Another factor (please specify): ________________ |
| Have you used ever used a service-finder tool, such as an online search platform, mobile app, or phone-based service like a HelpLine or provider directory to search for mental health treatment or related services? Please select all that apply. | 1. Yes, online search platform 2. Yes, mobile app 3. Yes, phone-based HelpLine 4. No, I have not used any service-finder tools |
| If you have ever used the following service search tools, please indicate your level of satisfaction with the tool.   - NAMI HelpLine - Anxiety and Depression Association of America (ADAA) Find a Therapist Directory - HelpWhenYouNeedIt.org - Psychology Today Find A Therapist Directory - SAMHSA Behavioral Health Treatment Services Locator | 1. I have not used this search tool 2. Have used, not satisfied 3. Have used, somewhat satisfied 4. Have used, very satisfied |
| Have you ever used a service-finder tool that is not listed above? If so, please list up to three. | Open text response |
| When you used a service-finder tool, did you experience any challenges or difficulties with the tool? Please select all that apply. | 1. The search tool was confusing to use 2. The search tool did not cover my specific service need 3. The search tool did not cover my geographic area 4. The search tool did not provide enough information 5. The information provided by the search tool was incorrect or out of date 6. I have used service search tools but did not experience any difficulties 7. I have not used any service search tools 8. Another difficulty (please specify): _________________________ |
| In general, please provide a comment on your overall experience with service-finder tools to search for mental health treatment and related services. | Open text response |
